# The Institutional Library

**Published:** 1915-11-06

**Authors:** 


### THE INSTITUTIONAL LIBRARY.

**Views on Some Social Subjects.** By Sir Dyce Duckworth, Bart. (London: George Allen and Unwin. 1915. Pp. 320. 7s. 6d. net.)

According to the publishers' announcement, "this volume consists of a collection of addresses and lectures" (which), "although they emanate from a physician . . . are not medical, but . . . express the views and opinions formed on many subjects by a medical mind . . ." This description, taken together with the title of the book, arouses expectations which are not verified by a perusal of Sir Dyce's collected orations. For in truth his essays, almost without exception, either are medical or touch the medical aspects of the topics dealt with. They are, as was to be expected, polished in style and dignified in expression. Profundity of thought is not apparent.

**Fatigue.** By Professor A. Mosso. Translated by Margaret Drummond, M.A., and W. B. Drummond, M.B., C.M. Third and Cheaper Edition. 1915. (London: George Allen and Unwin. Pp. 334. Price 2s. 6d net.)

This translation of Professor Mosso's well-known work first appeared in 1903, and is now issued in a cheaper form. It is not stated that any new matter has been incorporated, nor is there any obvious internal evidence of experiments or observations made within recent years. Mosso's careful work and experimental data of the 'seventies and 'eighties are still valuable, but they do not nowadays rank as quite the last words on the subject of fatigue.

**Urgent Surgery.** By Félix Lejars. Translated from the Seventh French Edition by W. S. Dickie, F.R.C.S., and E. Ward, F.R.C.S. Third English Impression. Volume II. (Bristol: John Wright and Sons, Ltd. 1915. Price 25s.)

The first volume of this important work on surgery was reviewed in the issue of The Hospital for December 12, 1914, and to the favourable terms then employed there is little need to add very much, as the second volume follows on naturally as a direct continuation of the first. The principal divisions of the subject-matter are apportioned between the two volumes as follows: In the first, head, neck, chest, spine, and abdomen; in the second, genito-urinary organs, rectum and anus, strangulated hernias, and the extremities. In each volume there are ten beautifully clear full-page plates, and the number of illustrations in the complete work total over a thousand. In a volume of this character the illustrations are of necessity a very important feature, and in this instance it must be admitted that Professor Lejars has been exceptionally fortunate in securing really clear and helpful drawings, together with about two hundred original photographs. As regards the translation, the excellence of which is an example of how such work ought to be done, it is interesting to note that this second volume has had to be completed by a hand other than that of Mr. Dickie, who was solely responsible for the conversion into English of the first volume. Dr. Ernest Ward took up the work when Mr. Dickie left for France with the Expeditionary Force, and has, at short notice, so efficiently completed the task that the reader will find it impossible to determine the point at which Mr. Dickie left off and Dr. Ward began. They have both succeeded in producing not merely a translation, but in preserving something of the French spirit and atmosphere. At this stage in the life of such a well-known surgical work it is hardly necessary to do more than reiterate the opinion that these volumes deserve to find an honoured place in hospital libraries, where they can be readily referred to by house surgeons and members of the visiting staff.

**Medical Annual Synoptical Index to Remedies and Diseases,** 1905 to 1914. (Bristol: John Wright and Sons, Ltd. 1915. Pp. 407. Price 8s. 6d.)

Those medical men who possess the series of *Medical Annuals* covering the last ten years will realise without explanation the advantages of also acquiring the "Synoptical Index" which has just been compiled by the same publishers. By the aid of this index it becomes possible rapidly to find a reference in any of the *Annuals* of those years instead of having to search volumes separately for the information required. Of the value of these *Annuals* to practitioners and members of hospital staffs it is needless to write; they have made themselves indispensable in private and institutional libraries. This new index, besides the innumerable references, contains a list of drugs with their doses in metric as well as in imperial measure according to the new edition of the British Pharmacopœia. It is interesting to observe that the Editor does not look with favour on the metric system as applied to dispensing, and advances, in a special article, cogent reasons for his opinion. Having regard to the scope of the *Medical Annual* this volume may be justly considered as practically an index to medical literature from 1905 to 1914 inclusive. It seems hardly necessary to have given the book a vivid scarlet binding which clashes with the familiar green of the *Annuals;* its size would have been sufficient for distinguishing purposes.

**Fighting the Fly Peril.** By C. F. Plowman and W. F. Dearden, M.R.C.S. Eng., L.R.C.P. Lond., D.P.H., J.P., Medical Officer of Health, Port of Manchester. With an Introduction by A. E. Shipley, Sc.D., F.R.S., Master of Christ's College, Cambridge. (London: T. Fisher Unwin. 1s. net.)

Recently Sir H. Johnston predicted that the wars of the future would be waged, not by nation upon nation, but by combined humanity upon the disease-carrying invertebrata, the flea, the mosquito, and the fly. Dr. Shipley, whose instructive book upon the danger of these insects, "The Minor Horrors of War," we discussed in The Hospital of July 3, points out, in his preface to the present volume, that, lacking scientific treatment, such a forecast would not be impossible, for the mosquito, he believes, undoubtedly contributed to the decline and fall of both the Greek and Roman Empires. The authors of the volume give a detailed account of the research work undertaken in America and England. The stronghold of the fly is the manure-heap, and the problem which the investigators have had to solve is the destruction of the larvæ in these heaps without diminishing the agricultural value of the manure. The account of the effects of the twenty-four chemicals tried in America, and the checking of the results by Mr. Dearden in England, should be read in full. Still, to attack the fly in its breeding season is a stupendous task when we consider its fecundity. The period of hibernation seems, at first sight, to offer better opportunities. Its hibernating habits are, however, still a controversial question, and future research must be directed to this important point. At present, therefore, we must fight the fly with the weapons in our possession. This book is well written and clearly illustrated, and the authors do well to emphasise that a systematic scientific campaign against the fly must not any longer be delayed.

**An Index of Treatment.** By Various Writers. Edited by Robert Hutchison, M.D., F.R.C.P., and James Sherren, F.R.C.S. Seventh Edition, Revised and Enlarged. (Bristol: John Wright and Sons, Ltd. 1915. Price 21s.)

Reference books of the type exemplified by the volume now under notice have of late years attained a considerable measure of popularity, as shown by the call for repeated editions. The reason is not far to seek. The general practitioner finds them very convenient for rapidly acquiring a concise knowledge of current views on the treatment of any given condition. The wideness of the range of the subjects dealt with in such publications naturally imposes a limitation on the comprehensiveness with which a particular subject can be treated, but, provided care is taken to keep pace with medical progress, there should be no difficulty in providing information of a really practical and helpful character in a moderate compass. The well-known "Index of Treatment," which is published by Messrs. John Wright and Sons, has now reached its seventh edition. The last revised edition is now just four years old, and, seeing that only a similar period elapsed between the publication of the first edition and the sixth, it cannot be said that this last revision has been undertaken with immoderate haste. The present edition has been thoroughly revised and several new articles added, among which may be mentioned those on the psychoneuroses, radium-therapy, and sterility. The surgical editorship has been undertaken by Mr. James Sherren in the place of Mr. Stansfield Collier. The number of the contributors is now larger than ever, while the book itself has had its bulk increased by 100 pages. As regards the subject-matter of the various articles, one cannot say more than that as the result of dipping here and there into the pages of this bulky volume, the information offered is at once practical, concisely stated, and based on the best of modern teaching. This "Index of Treatment" will undoubtedly continue to deserve the approval of the busy general practitioner.

**An Index of Prognosis and End-Results of Treatment.** By Various Writers. Edited by A. Rendle Short, M.D., F.R.C.S. (Bristol: John Wright and Sons, Ltd. 1915. Pp. 570. Price 21s. net.)

The general practitioner has many painstaking workers actively engaged on his behalf in compiling books of reference which shall assist him in the diagnosis and treatment of the exceedingly various conditions he is called upon daily to deal with. The result is that several excellent encyclopædias, indices of treatment and of symptoms are now available, and are apparently well appreciated. The volume now under consideration, although allied to such works, has an entirely different scope. Its aims cannot fail to appeal to practitioners, for the art of prognosis is vitally important in more senses than one, and it is extraordinarily difficult to gauge the end-results of treatment, even if one devotes much time and labour to searching the literature. The numerous contributors to this volume have striven to set forth the results of various methods of treatment in such a form as will enable the practitioner to obtain a fair opinion as to the prospects and risks of such treatment, and also they have endeavoured to furnish data by means of which the medical attendant may be able to predict with some show of accuracy what will happen to a particular patient. It may well be believed that the compilation of this volume has been an exceedingly laborious task. It has involved much search through the wilderness of modern medical literature and the following up of large numbers of patients both in hospital and in private practice. The information when obtained has to be very carefully sifted, for some records are not infrequently touched with *couleur de rose*. The usefulness of this work will be unchallenged; as a reference book it is unique. As the Editor remarks, "It will be for the future to improve upon the end-results of treatment here recorded which at the time of writing have attained thus far and no farther." No useful purpose would be served by attempting to appraise particular sections of this large volume; the list of distinguished contributors must be a sufficient guarantee that good work has been put into these pages, and that the more difficult problems connected with certain diseases have been handled as well as human experience allows. The necessary figures are lucidly tabulated and are so presented as not unduly to obtrude themselves. The publishers may well be congratulated on having added this volume to their previous successes, the "Index of Treatment" and the "Index of Differential Diagnosis of Main Symptoms."

**Feeble-Minded in Ontario.** Ninth Report. By Dr. Helen MacMurchy. (Toronto: 1915.)

We recently had occasion to write appreciatively of Dr. MacMurchy's summary of the work that is being done among the feeble-minded in Ontario. In this further report it is made clear that this work is being carried on effectively even under the present adverse conditions. The two most important events in connection with the feeble-minded in Ontario during the year are, according to Dr. MacMurchy, the passing of an Act which provides for the education of those children who, from any physical or mental cause, are unable to take advantage of any of the ordinary public or separate school courses, the only exception being that "persons whose mental capacity is incapable of development beyond that of a child of normal mentality at eight years of age" are not included in this definition; the second is the opening of a special clinic for the private examination of mentally-defective children and adults in the city of Toronto at the Toronto General Hospital.

The results of an investigation carried out by the Massachusetts Commission on the White Slave Traffic, which are noted in this report, are a striking indication of the necessity for the recognition of feeble-mindedness in girls at an early age, and also of the need for proper provision for their protection. "Three hundred women convicted of or arrested for prostitution were carefully studied and examined by experts. The results show that 154, or 51 per cent. were feeble-minded; 11 were insane, and 135 were rated as normal. Of the 135 rated as normal only a few ever read a newspaper or book, or had any real knowledge of current events, or could converse intelligently upon any but the most trivial subjects. Not more than six of the entire number seemed to have really good minds." Dr. MacMurchy reiterates the importance of avoiding half-measures in dealing with the feeble-minded as regards their supervision; "permanent care in a suitable institution is the only successful, economical, and humane method of dealing with mental defectives." The report also contains an excellent epitome of the work done in connection with the investigation and treatment of feeble-mindedness in England and in the United States of America, and it is comforting to be told that "steady progress is now being made all over the world" in the matter of the care of mental defectives.

